# Investigation of CCR7 Marker Expression Using Immunohistochemical Method and Its Association with Clinicopathologic Properties in Patients with Breast Cancer

**Published:** 2018-04-01

**Authors:** Laleh Vahedi, Maryam Ghasemi, Jamshid Yazdani, Samaneh Ranjbar, Banafshe Nouri, Ahad Alizadeh, Parvaneh Afshar

**Affiliations:** 1 Department of Pathology, School of Medicine, Mazandaran University of Medical Sciences, Sari, Iran; 2 Department of Biological Statistics and Epidemiology, School of public Health, Mazandaran University of Medical Sciences, Sari, Iran; 3 School of Medicine, Mazandaran University of Medical Sciences, Mazandaran, Iran; 4 Department of Epidemiology and Reproductive Health, Reproductive Epidemiology Research Center, Royan Institute for Reproductive, Biomedicine, ACECR, Tehran, Iran; 5 Research and Development Unit of Referral Laboratory, Deputy of Health Management, Mazandaran University of Medical Sciences, Sari, Iran

**Keywords:** Breast Cancer, CCR7, Clinicopathologic, Immunohistochemical staining

## Abstract

**Background:** Breast cancer is one of the most common cancers among women in the world, especially in Iran. There are large numbers of molecular and genomic factors causing breast cancer as well as many markers associated with tumor invasion.

Chemokines are small proteins that primarily regulate leukocyte trafficking in the homeostatic conditions and specific immune responses. Chemokine receptor 7 (CCR7) belongs a class A subtype 7-span transmembrane G-protein coupled receptor. CCR7 plays a role in the migration of tumor cells such as immune cells into lymphoid organs through binding to its only two ligands CCL19/CCL21.

High expression of this marker has been observed in breast cancer. However, there have been limited and contradictory data in studies conducted on the relationship between the increasing expression of this marker with various clinical and pathological factors.

**Materials and Methods: **This case-control practical study was carried out on total mastectomy samples from 70 patients with breast cancer and tumor-adjacent normal tissue using immunohistochemistry technique to assess the expression of CCR7 marker. The relationship among the marker expression with different clinical and pathological tumor factors such as age, tumor size, microscopic grade, neurovascular invasion, lymph node metastasis and tumor stage were evaluated in all patients. Since the both groups were matched for age, so McNemar test, Chi-square test and Fisher's exact test were used to compare the expression of CCR7 marker in the case and control groups. Conditional logistic regression was employed to compare the effects of other variables regarding the age harmonization.

**Results:** CCR7 expression was observed in 63 (91.4%) out of 70 studied patients and in tumor-adjacent normal tissue of 55 patients (78.6%), while the marker expression intensity in normal tissue was lower than tumoral tissue (P<0.032)

There was a significant relationship among the expression of CCR7 marker with disease stage (P<0.001), grade (P<0.035), lymph node metastasis (P<0.003), perineural invasion (P<0.037) and vascular invasion (P<0.01), but no significant relationship was found among CCR7 expression with other tumor clinicopathologic parameters such as age (P>0.19) and tumor size (P>0.105).

**Conclusion:** Increased expression of CCR7 has a significant relationship with disease stage, grade, lymph node metastasis and neurovascular invasion of breast cancer but has no relationship with age of patients and tumor size. Therefore, this biomarker can be utilized as a predictive factor for tumor metastasis and survival of patients.

## Introduction

 Chemokines are one of the prognostic factors in human breast cancer which play multiple roles in various cancer types, especially as a part of the inflammatory mediator networks. They act as angiogenesis factors and are important in the release of immune and tumor cells. The chemokines belong to a large family of small cytokines-like proteins that induce cytoskeletal rearrangement, adhesion to endothelial cells and targeted migration through interaction with G-protein coupled receptors ^10^.

CCR7 is a homeostatic chemokine receptor that is expressed in various subtypes of immune cells and is involved in their migration to the lymphoid organs. The CCR7 expression can be observed in naive immune cells, lymphocytes T and B, mature dendritic cells, natural killer (NK) cells and thymocyte subsets ^11^.

Recently, chemokines and chemokine receptors have been identified as key factors in metastatic process ^11^^, ^^12^. The chemokine receptor CCR7 is a 7-span transmembrane G-protein coupled receptor that can cause migration of cells to the secondary lymphoid organs by binding to their ligands (chemokines CCL19 and CCL21). CCL19 and CCL21 are expressed by stromal cells of primary and secondary lymphoid organs, endothelial cells of lymphatic vessels and peripheral tissues ^13^^,^^14^.

A sequence called DRY motif at the cytoplasmic end of transmembrane domain 3 (TM3) plays major role in controlling the CCR7 receptor activity and in coupling to G-protein. The presence of a polar reaction between arginine in DRY motif and glutamate in TM6 stabilizes inactive status of the receptor, referring to ionic lock ^15^.

The CCR7 expression has been reported in tumoral cells of different organs such as melanoma, breast, lung, prostate, head and neck, stomach and colorectal cancers as well as hematologic malignancies such as non-Hodgkin's lymphomas, which are involved in migration of tumoral cells to lymphoid organs such as immune cells ^16^.

The process of metastatic cancer is highly dependent on interaction between tumor and stromal cells. CCL21 could help to regulate tumor cell migration and invasion through CCR7 ^17^. It appears that CCR7-CCL21 axis in breast cancer is vital in lymph node metastasis ^18^^,^^19^.

Some studies conducted on the field of breast cancer have indicated that there is a significant relationship between the CCR7 expression and clinicopathologic properties in human breast cancer such as tumor size, histological grade and lymph node metastasis ^10^^, ^^13^^,^^20^^, ^^21^. However, other studies have shown that there is no significant relationship between the CCR7 expression and clinicopathologic properties in human breast cancer such as tumor size, patient’s age, tumor-involved lymph nodes and tumor grade ^22^. Also, in another study, the association was not positive in the tumor cells ^11^. The present study aimed to determine the CCR7 marker expression and its association with clinicopathologic characteristics using immunohistochemistry method in patients with breast cancer referred to Imam Khomeini Hospital, Sari, Iran between 2011 and 2016. 

Given the rising prevalence of breast cancer in different countries including Iran and the direct correlation of its prognosis with lymph node metastasis, lack of proper medical response, high mortality rate among females due to breast cancer and contrary results in this regard, it seems that conducting this study could help to resolve the existing conflicts and use of appropriate treatment in the future.

## MATERIALS AND METHODS

**Study type and participants**

In the present case-control and practical study, the CCR7 expression was investigated in the invasive tumor tissue of patients with breast cancer in the case group and in the tumor-adjacent normal tissue in the same slide of patients in the control group. This study was conducted to investigate the relationship between the CCR7 marker expression and clinicopathologic characteristics in the invasive tumor on the breast cancer tissue samples available in the archive of Pathology Department of Imam Khomeini Hospital, Sari, Iran between 2011 and 2016. 

The patients were divided into two groups of under 50 years and over 50 years. The tumor sizes were defined in three groups of 2 cm, 2-5 cm and above 5 cm.

**Inclusion criteria**

Study participants included patients diagnosed with invasive ductal carcinoma following the breast surgery and those who did not receive chemotherapy before surgery.

**Exclusion criteria**

The tumors with unsuitable blocks or slides for immunohistochemical or giemsa staining and also patients with incomplete clinicopathologic information were excluded from the study. Each study group consisted of 70 patients.

**Data collection**

At first, the samples not exposed to chemotherapy and radiotherapy were collected from the archive of Imam Khomeini Hospital, and then the questionnaire was completed.

Then, required paraffin blocks were taken out from the archives and the slides stained with hematoxylin-eosin were prepared from the invasive tumoral and normal tissues (including apparently tumor-adjacent normal tissue) as controls.

Microscopic parameters such as lymphatic invasion, vascular invasion, the level of tumor differentiation and the presence of tumor were investigated. 

In immunohistochemistry method, at first 4-micron sections of the selected block were placed on slides of Saylyn S3003 and in 60ºC for 1 hour, and then were exposed to xylol, absolute ethanol and ethanol 96° (twice in each solution and for 5 min in each time) in three steps for deparaffinization and at last were washed by the running water. After drying, the slides were transferred to 1% hydrogen peroxide mixture (to eliminate internal peroxidase) and methanol followed by target solution after 10 min. Next, they were placed in an autoclave under pressure of 105 MmHg/h20 for 13 min to reach the boiling point. Afterwards, the microwave power was decreased up to 40%, then the tissues were removed after 15 min and left to reach room temperature. After rinsing with running water and wash buffer, the margins of tissues were determined using Dako stylus and they were placed in a moist chamber in the dark. The samples were covered with Anti-CCR7-antibody marker (1:1000 dilution) and placed in the refrigerator for 18 hours. The positive control was splenic tissue and negative control was PBS (peripheral blood smear). The control tissue was the tumor-adjacent normal tissue. In order to examine the specificity of immune staining, both positive and negative controls were run at the same time in each experiment. In the next stage, the samples were left in an envision environment for an hour at room temperature, and then washed twice with wash buffer. The DAB solution was poured on slides; if a brown color change appeared after 1-2 min, they would place again in wash buffer for 2 minutes. Afterward, the slides washed by distilled water were stained with Mayer's hematoxylin, rinsed in distilled water again and fixed in xylol. Finally, the slides were mounted with Entellan. The prepared slides were examined and reported by two experienced pathologists in the field of CCR7 marker. The expression pattern of CCR7 marker was in cytoplasmic type and brown cytoplasm was considered as positive. All slides were studied in terms of the extent and intensity of staining and the percentage of stained cells and the results were reported as a semi-quantitative ^2^. The staining index of CCR7 marker was calculated via multiplication (staining index), which is determined by multiplying the score for intensity of cell staining by the score for proportion of stained cells. The intensity of cell staining was reported as follows:

Negative = 0 

Weak brown positive = 1

Moderate brown positive = 2 

Strong brown positive = 3

The percentage of stained cells was also reported as follows: None staining 1: 1-25%, 2: 25-50%, 3: 50-75% and 4: >75%.

Staining index ≥6 was considered as an expression at high intensity and <6 as an expression at low intensity (10). This marker was prepared from the ABCAM Co. in the U.S.A.

**Statistical analysis**

Initially, descriptive statistics such as mean ± standard deviation for age, frequency tables, ratios and percentages were applied for other variables to describe the obtained data. Given that both case and control groups were matched by age, McNemar, chi-square and fishers exact tests were used to compare the CCR7 expression. According to matched age, conditional logistic regression was used for assessment of variables. Data were analyzed by STATA12 software and P-value<0.05 was considered as statistically significance level.

## Results

Totally, 70 patients with breast cancer were enrolled and evaluated in the present study. The clinicopathologic findings of patients are briefly given in Table 1.

**Table 1:** Clinicopathologic findings of patients with breast cancer

Percentage	Number		
44.3	31	<50years	Age
55.7	39	>50years	
22.9	16	>2cm	Tumor size
57.1	40	2-5cm	
20	14	>5cm	
22.9	16	1	Histologic grade
62.9	44	2	
14.3	10	3	
44.3	31	Yes	Perineural invasion
55.7	39	No	
47.1	33	Yes	Vascular invasion
52.9	37	No	
60	42	Yes	Lymph node metastasis
40	28	No	
14.3	10	1	Disease stage
38.6	27	2	
47.1	33	3	

**Fig 1:**
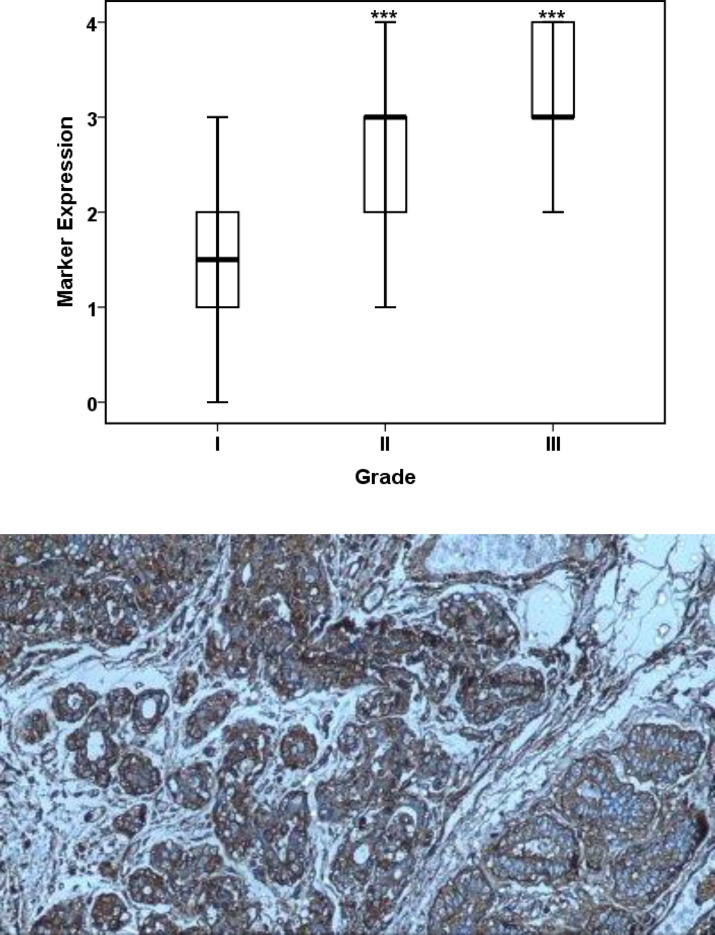
Positive staining of CCR7 marker in cytoplasm of breast cancer cells and moderate staining of this marker in normal breast tissue cells (100X)

**Fig 2:**
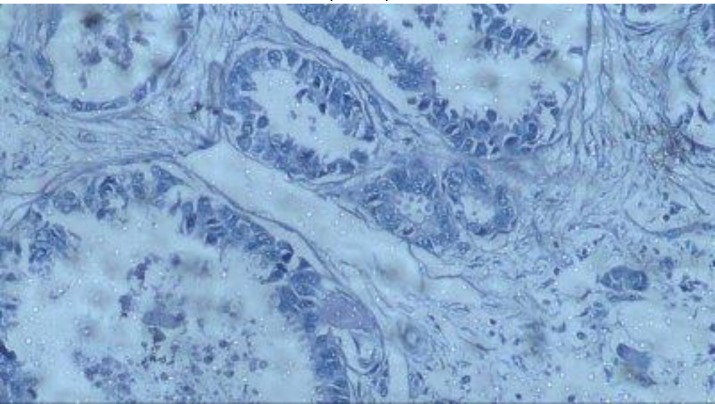
Negative staining of CCR7 marker in cytoplasm of breast cancer cells by immunohistochemical staining (100X)

**Fig 3:**
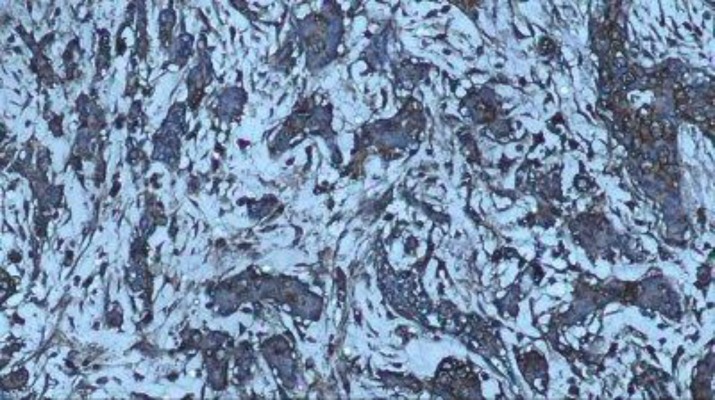
+1 (Weak) staining of CCR7 marker in cytoplasm of tumor cells in breast carcinoma by immunohistochemical staining (100X)

**Fig 4:**
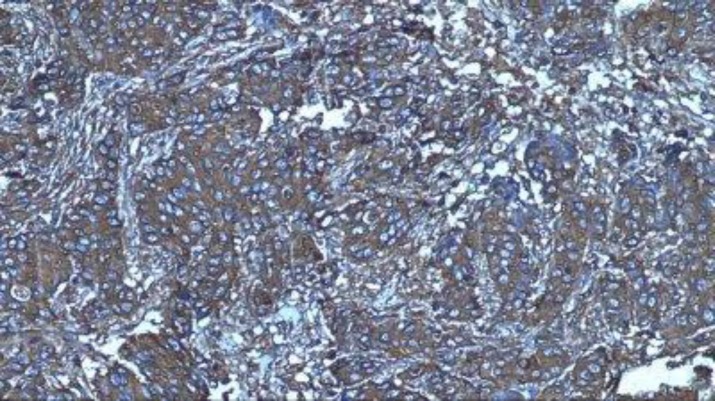
+2 (moderate) staining of CCR7 marker in cytoplasm of tumor cells in breast carcinoma by immunohistochemical staining (100X)

**Fig 5:**
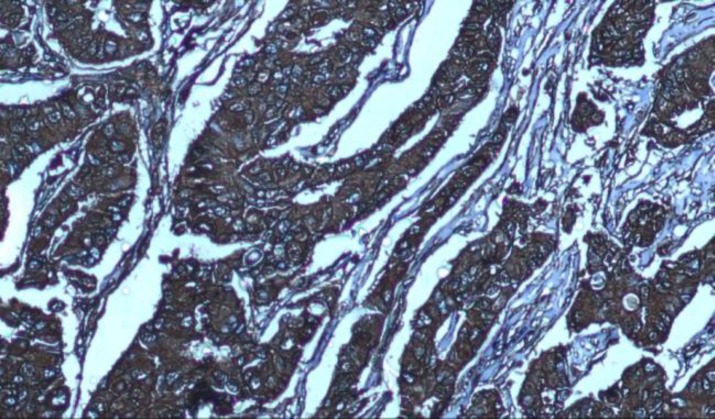
+3 (Strong) staining of CCR7 marker in cytoplasm of tumor cells (100X)

After immunohistochemistry staining, the CCR7 marker expression and intensity of staining were compared in the two groups (Table 2). In the study group, 64 patients (91.4%) had positive staining and 6 patients (8.6%) had negative staining, but, in the control group, 55 patients (78.6%) had positive staining and 15 patients (21.4%) had negative staining. There was no statistically significant correlation in the CCR7 expression between the two groups (p>0.064), but the intensity of CCR7 expression in the study group was significantly more than control group (P<0.032). In other words, the intensity of marker expression in invasive tumor tissue was more than in tumor-adjacent normal tissue. The expression level and intensity of CCR7 in the two groups have been presented in Figures 1 and 2. Since the control group was chosen out of tumor-adjacent normal tissues and both groups were matched for age, so Chi-square test was used to analyze this section and compare the CCR7 marker expression in the two groups. The intensity of staining is given in Figures 1 to 5.

**Table 2:** Comparison of staining intensity of CCR7 marker in the two case and control groups

Severity of Expression						
					Group	
	Frequency			Percent		
	Control		Case	Control		Case
Negative	15		6	21.4		8.6
Weak	31		13	44.3		18.6
Moderate	23		28	32.9		40
Strong	1		23	1.4		32.9
Total	70		70	100		100

**Table 3:** Comparison of staining index (SI) of CCR7 marker in the two case and control groups

	Staining index< 6	Staining index< 6
Case group	48%	52%
Control group	89%	11%

Investigation of marker expression in the three grades demonstrates that the severity of marker expression is significantly elevated by increasing the grades of disease. A more detailed assessment indicates that grades one and two are significantly different in terms of marker expression, but there is no statistically significant difference between grades two and three (Table 4).

**Table 4:** Investigation of relationship between CCR7 expression and grade in patients with breast cancer

**Percentiles**									
		Grade	Percentiles						
			5	10	25	50	75	90	95
Weighted Average (Definition 1)	Marker Expression	I	0	0	1	1.5	2	2.3	0
		II	0	1	2	3	3	4	4
		III	2	2	2.75	3	4	4	0

Following results were obtained by comparing the CCR7 marker expression with clinicopathologic parameters of patients with breast cancer and statistical analysis of the data by MCNemar and Fisher’s exact tests.

There was no direct and significant relationship between tumor size and marker expression (Figure 3).

The correlation between the grade and the stage of disease and the marker expression indicates a strong direct correlation between these two variables (Figures 4 and Figures 5).

The marker expression in patients with vascular involvement, positive perineural and lymph node metastasis was significantly more than in patients with negative index (Table 5).

Investigating the correlation between age and marker expression demonstrates that aging has no significant effect on the expression of studied markers (Table 5).

The relationship between clinicopathologic parameters of patients and the CCR7 marker is listed in Table 5.

**Table 5:** Relationship between CCR7 expression and clinicopathologic characteristics of breast cancer

**Clinicopathologic parameters**		**CCR7 expression**				**P value**
		**Positive**		**Negative**		
		**Number**	**Percentage**	**Number**	**Percentage**	
Age	>50	37	52	4	5.5	0.19
	≤50	27	40	2	2.6	
Tumor size	>2cm	13	18.57	3	4	0.105
	2-5cm	37	52.85	3	4	
	>5cm	14	20.6	0	0	
Histological grade	1	13	18.57	3	4	0.035
	2	41	58.97	3	4	
	3	10	14.0	0	0	
Perineural invasion	Yes	31	44.3	33	47.2	0.0375
	NO	0	0	6	8.5	
Vascular invasion	Yes	33	47.2	31	44.3	0.018
	NO	0	0	6	8.5	
Lymph node metastasis	Yes	42	60	22	31.5	0.003
	NO	0	0	6	8.5	
Disease stage	1	6	8.57	4	5.7	0.001
	2	25	35.37	2	2.8	
	3	33	47.2	0	0	

## Discussion

 Lymphatic vascular endothelial cells of peripheral tissues and stromal cells of lymph tissues produce two types of chemokine ligands, CCL19 and CCL21, which couple to the CCR7 protein receptor. It is believed that this receptor is produced by dendritic cells, B-lymphocytes, T memory cells and naive cells^1^. 

The CCR7 biomarker in many animal studies, in vitro condition and in human researches is presented as prognostic factor. The expression of this biomarker can predict the existence and amount of metastasis in breast cancer ^2^.

Increased expression of CCR7 receptor has been reported in lung cancer ^3^, non-small cell, esophageal cancer ^4^ and stomach cancer ^3^.

Upregulation level of CCR7 marker has been reported in head and neck tumoral squamous cell ^5^^,^^6^, metastatic tumor cells and regional lymph nodes. The association between CCR7 markers and lymph node metastasis in the patients with thyroid and colorectal cancer was also found ^7^^-^^9^.

In the study by Liu et al. on 200 samples of invasive ductal breast cancer, the CCR7 marker expression was reported 82%. ^10^. Fei li et al. examined 60 patients with primary breast cancer and found CCR7 marker expression (60%) with high intensity ^13^. In the study of Philippe A Cassier et al., CCR7 marker was not positive in tumor cells of breast cancer but was positive in 43% of spindle-shaped stromal cells^11^. In the present study, the CCR7 expression level was 91% among the patients. The difference in the measured levels can be due to different methods of staining or assessing the CCR7expression.

Fei li et al. ^13^ and Liu et al. ^10^ demonstrated no significant correlation between patients’ age and CCR7 marker expression. Similar results were obtained in the present study and no significant relationship was found.

Liu et al. ^10^ reported a significant correlation among the incidence of CCR7 marker, lymph node metastasis and disease stage. Fei li et al. ^13^ also showed that there is a significant relationship among the CCR7 expression, lymph node metastasis and clinicopathologic stage. N cabioglu et al. ^21^ in a study on 197 breast cancer patients with stage T1 proved that cytoplasmic expression of CCR7 with high intensity in tumors with lymph node metastasis is higher than in tumors without lymph node metastasis (p=0.013). The results of our study showed that the incidence of CCR7 marker has had a significant role in breast cancer as a prognostic biomarker in metastatic lymph nodes. In other words, there was a significant correlation among the CCR7 expression, level of lymph node metastasis and disease stage. Liu et al. ^10^ found that the highest CCR7 marker expression (71%) was in patients with stage II, while the highest expression (47.2%) in the current study was observed in patients with stage III. 

Concerning the relationship between the level of marker expression and grade, Liu et al. ^10^ showed a significant association, so that the majority of the patients were in grade II. However, Andre et al. reported no significant correlation between the grade and level of CCR7 expression ^22^. Our study has shown a significant relationship between them and the majority of our patients were in grade II.

N Cabioglu et al. ^21^, Andre et al. ^22^ and Liu et al. ^10^ found no significant relationship between tumor size and CCR7expression. In the study by Liu et al^10^, 54% of patients were in the category of 2-5 cm. In our study, there was no significant statistical correlation between these two and 52% of patients had tumor size between 2 and 5 cm.

In the study by Hernandez et al ^25^, no significant correlation was found between CCR7 expression and vascular involvement, but CCR7 expression in our study was correlated with vascular and perineural invasions.

Some of the key limitations of this study included the short-term follow-up, study of distant metastasis and survival rate of the patients.

## CONCLUSION

 The results obtained in this study demonstrated the presence of a strong relationship between CCR7 expression and most of the clinicopathologic properties of breast cancer, including lymph node metastasis, tumor grade, disease stage, perineural involvement and vascular invasion, whereas no significant correlation was found between age and tumor size. Therefore, this biomarker can be used as a prognostic marker for predicting metastasis. In order to achieve greater certainty as a result, the investigation of the relationship among the survival of patients, distant metastasis and recurrence rate with CCR7 marker expression is highly recommended as a subject for future studies. 
